# Older adults report moderately more detailed autobiographical memories

**DOI:** 10.3389/fpsyg.2015.00631

**Published:** 2015-05-19

**Authors:** Robert S. Gardner, Matteo Mainetti, Giorgio A. Ascoli

**Affiliations:** ^1^Center for Neural Informatics, Structures, and Plasticity, Krasnow Institute for Advanced Study, George Mason UniversityFairfax, VA, USA; ^2^Psychology Department, George Mason UniversityFairfax, VA, USA; ^3^Molecular Neuroscience Department, George Mason UniversityFairfax, VA, USA

**Keywords:** autobiographical memory, memory content, aging, forgetting, recollection, episodic memory, word-cue technique

## Abstract

Autobiographical memory (AM) is an essential component of the human mind. Although the([A-z]+) amount and types of subjective detail (content) that compose AMs constitute important dimensions of recall, age-related changes in memory content are not well characterized. Previously, we introduced the Cue-Recalled Autobiographical Memory test (CRAM; see http://cramtest.info), an instrument that collects subjective reports of AM content, and applied it to college-aged subjects. CRAM elicits AMs using naturalistic word-cues. Subsequently, subjects date each cued AM to a life period and count the number of remembered details from specified categories (features), e.g., temporal detail, spatial detail, persons, objects, and emotions. The current work applies CRAM to a broad range of individuals (18–78 years old) to quantify the effects of age on AM content. Subject age showed a moderately positive effect on AM content: older compared with younger adults reported ∼16% more details (∼25 vs. ∼21 in typical AMs). This age-related increase in memory content was similarly observed for remote and recent AMs, although content declined with the age of the event among all subjects. In general, the distribution of details across features was largely consistent among younger and older adults. However, certain types of details, i.e., those related to objects and sequences of events, contributed more to the age effect on content. Altogether, this work identifies a moderate age-related feature-specific alteration in the way life events are subjectively recalled, among an otherwise stable retrieval profile.

## Introduction

Autobiographical memory (AM) refers to the recollection of personally experienced episodes specified in time, and has critical functions among adults of all ages (e.g., Pillemer, 1992; Bluck et al., 2005; Bluck and Alea, 2011; Waters, 2014). As any given AM is associated with a unique episode retrieved from a countless variety of experiences, any two AMs may differ greatly in terms of the type and amount of detail they contain (i.e., their content). For example, some memories contain a high degree of sensory and spatial information, whereas others comprise little sensory information but instead are associated with distinct thoughts and feelings; at the same time, certain memories are highly vivid and contain many details from numerous content categories, while others contain few and scattered elements.

The type and amount of subjective detail remembered in AM appear to be important dimensions of recall. For example, AM content is proposed to play an important role in source monitoring (i.e., the process that determines the association between a memory and a particular context or source; Johnson and Raye, 1981; Johnson et al., 1988; Hashtroudi et al., 1990). As accurate source determinations may deteriorate in older adults (e.g., Cohen and Faulkner, 1989; Hashtroudi et al., 1990; Johnson et al., 1993; Gerlach et al., 2014), an understanding of age-related changes in AM content is essential. Nonetheless, quantitative characterization of AM content is lacking, most notably across a range of ages representative of the adult population, and across the life span of a given individual.

Autobiographical memory content has been probed in several ways. Some studies have assessed the veridicality of AMs by contrasting recalled details with verifiable event information. Resulting findings have highlighted the constructive and subjective nature of AM and suggest that the details retrieved by an individual may not match the event as it transpired in the material world (see e.g., Bartlett, 1932; Conway and Pleydell-Pearce, 2000). As such, many studies have focused on AM content as constructed and reported by the individual. Subjective memory content is typically measured by participant report using ordinal scales. For example, the Memory Characteristics Questionnaire (Johnson et al., 1988), Autobiographical Memory Questionnaire (Rubin et al., 2008), and Memory Experiences Questionnaire (Sutin and Robins, 2007) rate the perceived amount (or clarity) of sensory (e.g., visual, auditory), spatial, temporal, and emotional detail associated with a particular memory. Likewise, perceived event specificity has also been rated (e.g., Kopelman et al., 1989; Piolino et al., 2002; Sutin and Robins, 2007).

Relatively few studies have reported counts of the number of subjective details retrieved in AMs (Berntsen, 2002; Levine et al., 2002; St. Jacques and Levine, 2007; Addis et al., 2008, 2010); these studies typically use standard sets of cues (e.g., event-cues) to elicit memories, and subsequently collect from the participant written or spoken narratives of recalled experiences. Experimenters process each narrative (e.g., segment unique AMs), and score each memory for content. Several experiments using these techniques have shown that older (as compared with younger) subjects report fewer episodic details contained within memory for unique life experiences (e.g., Levine et al., 2002). However, AM content analyses have largely been confined to few and restricted life periods and age groups, and to memories that may not reflect naturalistic recall, e.g., those recalling experiences simulated in the laboratory (Hashtroudi et al., 1990) or those elicited using typical life event cues (Levine et al., 2002).

We previously introduced the Cue-Recalled Autobiographical Memory test (CRAM; see Gardner et al., 2012), in part, to address these limitations. CRAM elicits AMs using a modification of the word-cue technique (Crovitz and Schiffman, 1974). In contrast to traditional methods, word-cues are generated based on their usage frequency in spoken and written language in order to emulate naturalistic cues. Therefore, elicited AMs should be more closely matched to those recalled in everyday situations. Participants subsequently identify the age of each AM, and count the number of details recalled within specified features (e.g., temporal detail, spatial detail, persons, objects, emotions, temporally linked events, and other contextual elements) similar to those used in previous designs (e.g., Johnson et al., 1988; Hashtroudi et al., 1990; Levine et al., 2002). Given CRAM’s reliance on participants to specify what constitutes a detail within a feature category, this technique permits efficient data collection. This key advantage enables collection of larger data sets and relatively comprehensive AM coverage across the life span of numerous age groups. In addition, collecting count data (as opposed to relying on ordinal scales) facilitates interpretation of between-subjects and between-groups comparisons.

Despite its methodological differences (as compared with previous tests developed to probe AM), CRAM reliably reproduces several results of prior studies among young adults. For example, AMs cued by CRAM produce temporal distributions which completely replicate characteristics of those produced by traditional techniques, e.g., the retention interval and childhood amnesia (Rubin, 1982, 2000; also see Rubin et al., 1986; Rubin and Schulkind, 1997; Janssen et al., 2011). Moreover, AMs scored by CRAM show a temporal decay in content, a reported component of AM retrieval (e.g., Levine et al., 2002; Piolino et al., 2002; Janssen et al., 2011).

The current work builds on this prior research, which focused on college-aged subjects, by applying CRAM to individuals of various ages across the adult life span (18–78 years old). We utilized both in-person and Internet-based testing^1^ to further enhance data collection. The resulting data provide numerical counts of AM content from a diverse subject pool that should expand our understanding of the relationship between aging and subjective recollection.

In particular, this research was conceived to describe age-related changes in subjective AM content, and to test the hypothesis that, on average, subjective reports of AM detail decrease both with the age of the event and with the age of the subject. Similar to studies that quantitatively described age-dependent modulation of the temporal distribution of AM retrieval (Rubin et al., 1986; Rubin and Wenzel, 1996; Rubin and Schulkind, 1997; Rubin, 2000) this work quantitatively characterizes age-dependent modulation of feature-specific recollection.

Application of CRAM to older subjects may also contribute to AM theory. The reminiscence bump is an increase in retrieval of AMs pertaining to episodes from adolescence to early adulthood and is most clearly observed in older adults (see Rubin et al., 1986, 1998; Jansari and Parkin, 1996; Janssen et al., 2005, 2011). Previous studies show that AMs from the bump, as collected using the word-cue technique, are not associated with enhanced phenomenological characteristics of recollection, e.g., vividness or re-living (Rubin and Schulkind, 1997; Janssen et al., 2011). However, whether content counts of these memories correlate with their retrieval probabilities remains an open question. For example, it is possible that memories rich with detail have relatively high association probabilities with a given memory cue, causing these AMs to be frequently accessed; that is, enhanced retrieval of bump memories may produce (or result from) enhanced content retrieval. More specifically, this research tests the hypothesis that subjective content reported from bump AMs is increased compared with that reported from non-bump AMs. In addition, this approach and resulting data may be useful to inform theories of memory, e.g., multiple trace theory (Nadel et al., 2000).

## Materials and Methods

### The Cue-Recalled Autobiographical Memory Test

The Cue-Recalled Autobiographical Memory test is a computerized interactive test presented in web-browser format. It collects counts of the number of details (elements) within categories (features) that compose naturalistically word-cued AMs dated to specific life periods. Complete specifications of the test and instruction provided to participants have been previously reported^2^ (for full detail see Gardner et al., 2012). Here, each section of the test is briefly described.

Prior to eliciting AMs, CRAM collects demographic information for each participant. Subjects are then presented the following definition of AM and subsequent instruction:

“Autobiographical memories are recollections of past episodes directly experienced by the subject. These memories should be of a brief, self-consistent episode of your life. An episode can be as short as a single snapshot and up to a few seconds long. … If the memory you think of refers to a typical and repeated episode that happened regularly or multiple times in your life, you can use it only if you can fixate on a specific individual event. If you can only recall the generic (repeated) event, look for another memory.”

The test is designed to probe memory of unique personally experienced episodes, for example, memory for a conversation one had with friends at a recent dinner party, or memory of meeting one’s spouse. This type of memory is considered episodic and has been frequently contrasted with memory for factual information about the world or oneself (semantic: Tulving, 1972, 1985).

Naturalistic word-cues are then presented to elicit memories. The participant reads through a list of seven words and labels the first recollection retrieved, for subsequent identification. Subjects are further instructed that the cued AM does not necessarily have to relate to any one word or to the entire list of words, but rather is “the first autobiographical memory that the words bring to mind.” The list of word-cues is randomly selected from the British National Corpus^3^, a compilation of 100 million written and spoken words (see Gardner et al., 2012 for word processing details). Thus, this procedure provides cues that are presented proportionally to word frequencies observed in everyday settings. For example, the word “waiting” appears in the corpus 474 times whereas the word “yard” appears 71 times. Therefore, “waiting” is ∼6.7 times more likely than “yard” to be presented as a word cue. As the words are sampled randomly from the corpus each time a list is generated, the specific words selected may be dramatically different for any two presentations within and across subjects.

Once AMs are cued and labeled, participants are presented with their AM labels one by one to date each memory. Specifically, the participant places each memory into one of 10 temporal bins, which segment his or her life span into 10 equal intervals (a similar binning procedure has been used previously: see McCormack, 1979; Howes and Katz, 1992; Gardner et al., 2012). If necessary, up to three temporal bins could be assigned to a single AM. To increase dating accuracy, the temporal range associated with each bin (i.e., the cutoff values computed from the subject’s age) is presented in terms of time from the present, age of the participant, and month and year. However, as the size of a given temporal bin increases linearly with the participant’s age at the time of testing (e.g., the size of a bin for a 20 year old is half that for a 40 year old), this procedure also reduces the temporal resolution associated with a memory in older (relative to younger) subjects.

The age of the participant at the time of a recalled event is estimated as the mid-point of its assigned temporal bin. The retrieval probability of an AM falling within each bin or age range is computed by dividing the number of AMs dated to each temporal period by the total number of dated AMs. Collectively, these measures are used to construct the temporal (life span) distribution of AMs. In this work, Recent AMs are defined as memories of events that occurred within the most recent 10 years of life; Remote AMs are defined as memories of events that occurred more than 10 years from the present moment. Overall, ∼4% of AMs were assigned to more than one temporal bin (presumably resulting from a lack of confidence that the AM occurred within the temporal range associated with a single bin). This proportion mildly increased for Remote AMs (Remote: 5.0%; Recent: 3.7%, *p* < 0.001), suggesting a reduction in a given subject’s confidence to date these older episodes. In addition, younger subjects dated AMs to multiple bins slightly more frequently (4.0%) than older subjects (3.0%; *p* < 0.05), potentially due to the age-dependent nature of a given bin’s temporal interval size (the temporal range associated with a bin increases proportionally with the subject’s age at the time of testing).

After AMs are dated, participants are once again presented with their AM labels to score the content associated with a selected memory. In particular, participants are instructed to count the number of details remembered within each of eight categories, i.e., *Things* (objects), *Feelings* (emotional details), *People* (unique individuals), *Places* (spatial details), *Times* (temporal details), *Episodes* (temporally linked events), *Contexts* (other contextual details), and *Details* (all remaining details, including actions). Participants provide counts of details rather than event descriptions. The order in which each category is presented is randomized for each person but fixed across all AMs for a given individual. The exact definitions of these eight categories were previously reported (Gardner et al., 2012) and are available at http://cramtest.info. In addition, CRAM provides, through clickable links, additional examples of what constitutes a detail within a given category and general scoring guidance. Every detail category is called a “feature,” each reported detail associated with a given feature is referred to as an “element,” and the summed number of elements across all features for a given memory is called “total content.”

### In-Person and Internet Testing

The Cue-Recalled Autobiographical Memory test was completed locally under experimenter supervision or remotely over the Internet^4^. From each subject who completed testing in person, 30 AMs were cued and dated, of which a subset of 10 was scored for content (see Gardner et al., 2012; the first two AMs were considered practice and not analyzed). AMs were selected for scoring to maximize life span coverage in the entire dataset. Specifically, a single AM was scored from each life span bin represented by a participant. From the participant’s remaining AMs, memories were selected in order from the least to most represented bin (in the entire dataset across all participants) until 10 memories were scored in total; only for an initial sample of subjects (*n* = 110) the number of scored AMs was instead a function of the number of temporal bins represented.

In contrast to in-person testing, CRAM’s online protocol offers subjects the choice of several test options (i.e., Atomic, Mini, Extended, and Full tests) which differ according to the number of AMs cued, dated, and scored. These options are included to promote test completion by suiting a wide range of subjects who may vary in their commitment and eagerness to participate. The Atomic test cues one AM which is dated and scored for content. At the end of the Atomic test, subjects are asked if they would like to complete the Mini or Full test. The Mini test cues five AMs, which are each dated and scored. At the end of the Mini test, subjects are invited to extend the test. If a subject agrees, an additional fifteen AMs are cued and dated, five of which are scored; this option is categorized as the Extended test (which in total cues and dates 20 AMs, and scores 10 for content). The Full test cues 20 AMs, each of which is dated (only nineteen subjects completed instead a different Full test version which cued and dated 30 AMs as outlined in the in-person protocol). Subsequently, a subset of 10 memories is scored for content. Memory selection for scoring in the Full and Extended tests follows the same rules as those for in-person testing. Given these selection rules, the proportion of scored AMs in a particular life span interval may differ from that typically retrieved. Thus, when presenting aggregate measures of content (i.e., those within a given subject group) not restricted to a particular life period, content values across dating bins are weighted according to the applicable AM temporal distribution.

During online data collection, participants are encouraged to complete the Full test (or Extended test, if opting initially for the Mini test). This is accomplished by pre- and post-test advertisement for the opportunity to explore an interactive summary report of one’s results with the ability to make direct comparisons with results from specified age ranges, solely after completion of the Full (or Extended) test. We stress that, despite their variety (e.g., in duration), all test types provided subjects with the same instruction on AM classification, cueing, dating, and scoring (which were also identical to in-person testing).

When conducting analysis on an individual level, we assumed that a unique test ID corresponds to a unique user. By and large this assumption should be valid; however, it is likely that a subset of users took the test multiple times and were assigned distinct user IDs on each occasion. Moreover, undertaking the Mini or Full version of CRAM following completion of the Atomic format was not coded in the same way as extending the Mini test; in particular, those who opted for the Atomic test and subsequently decided to undergo longer testing were assigned a new user ID. We emphasize that those users who were interrupted or opted to take a break mid-test, however, maintained a single user ID. Likewise, users who opted to extend the Mini test were categorized as taking the Extended test and assigned one unique user ID. As data collected prior to and subsequent to the decision to extend the test was equivalent (data not shown) it appears that practice and in-depth knowledge of dating and scoring procedures did not influence retrieval as measured by CRAM. Unless indicated otherwise, data were collapsed across testing conditions and test types.

### Participants

As CRAM is freely accessible online^5^ and indexed by popular search engines, data are continuously collected from Internet-browsing individuals. To supplement these unsolicited data, additional individuals were actively recruited from the undergraduate population of George Mason University (GMU), from GMU staff and faculty, and from the local community, obtaining in all cases informed consent. With the exception of undergraduates, recruited subjects were given the choice to complete testing locally at GMU with a researcher present or remotely over the Internet. Recruited undergraduates (ages 18–36 years old) invariably completed the study for course credit and took the test under experimenter supervision; these data from recruited students have been reported previously (Gardner et al., 2012) and included here to best estimate AM content in relatively young subjects. However, the amount of data collected from this age range was substantially augmented by the current approach (exclusively through online testing) almost tripling the previous sample of scored AMs (when pooled together) from these younger subjects. No identifiable personal data were stored. All recruitment and testing procedures were approved by the GMU institutional review board.

In total, 17,482 AMs were dated from 2,561 unique test IDs (Mean Age = 34 years old, SD = 14, range: 18–78 years old; 67% female; 81% native English speakers). A subset of subjects (*n* = 640) dated AMs, but did not score their content, and were restricted to memory dating analysis. In addition, content measures from 560 Internet-collected AMs (spanning 192 unique test IDs) were inadvertently overwritten prior to back-up, and thus these AMs were also restricted to dating analysis (complete content measures from a subset of AMs from four of these 192 subjects were retained, however, and included in content analysis). After accounting for these events, a total of 6,492 AMs were scored for content (76% scored online) from 1,733 subjects. Fifty-two percent of Internet-scored AMs were collected from the Full test, 20% from the Atomic test, 16% from the Mini test, and 12% from the Extended test; subject age was equivalent across test types (*p* > 0.10).

### Data Screening

Data were further inspected to identify data entry errors or otherwise lazy and inauthentic reporting (e.g., see Gardner et al., 2012). Positive cases were removed from analysis. For example, AMs were excluded if a subject reported an identical number of elements for each of the eight features. In addition, all AMs were removed from seventeen participants whose scoring across the majority of their AMs reflected this pattern (201 AMs in total). Memories were also excluded from two participants who reported either 1 or 11 elements in each feature category across all scored AMs (14 AMs), and from two subjects who reported unique scores for each feature but identically scored all memories (15 AMs). Altogether, these exclusions totaled 232 scored AMs.

Subsequently, extreme total content values were identified as those greater than three times the Inter-Quartile-Range (IQR) above the 75th percentile within a given age range. Data meeting this criterion were considered outliers and excluded from analysis. This procedure was performed separately for AMs collected from each of the following age ranges: 18–25; 26–35; 36–45; 46–55; 56–65; 66–78 years of age. The outlier threshold ranged from 72 to 98 elements per memory depending on the age group (18–25 years old: 72 elements; 26–35 years old: 79 elements; 36–45 years old: 91 elements; 46–55 years old: 98 elements; 56–65 years old: 94 elements; 66–78 years old: 80 elements). This step resulted in the removal of 223 AMs: 78 AMs were excluded from 18 to 25 year old subjects (3.0% of the total within this age range), 52 AMs (3.5%) from 26 to 35 year old subjects, 39 AMs (4.0%) from 36 to 45 year old subjects, 33 AMs (5.1%) from 46 to 55 year old subjects, 16 AMs (4.5%) from 56 to 65 year old subjects, and 5 AMs (2.1%) from 66 to 78 year old subjects. **Table 1** provides a summary of the number of scored AMs ultimately retained for data analysis across test types and participant groups.

**Table 1:** The distribution of scored autobiographical memories (AMs) collected across test types and participant groups.

	Younger subjects (18–45 years old)					Older subjects (46–78 years old)				
	In-person	Atomic	Mini	Extended	Full	In-person	Atomic	Mini	Extended	Full
*n* (Subjects)	188	699	122	58	203	6	211	47	21	69
Mean age	20.9	29.6	30.3	30.7	29.5	53.5	55.8	56.3	54.4	57.8
SD age	4.3	7.7	7.5	8.0	7.5	10.8	7.2	7.1	6.3	9.0
% Female	76	71	78	84	67	83	63	66	86	64
% Native	73	80	81	84	83	100	90	87	86	87
*n* (Memories)	1489	699	515	409	1747	45	211	201	147	574

*The number of subjects and memories included in content analysis is shown as *n*. Percent Native indicates the percent of subjects who are native English speakers. SD, standard deviation.*

### Statistics

Analyses were primarily conducted using memory as the unit of observation. Findings were corroborated using an individual subject level approach (see Results). Binary logistic regression was run to assess the effects of participant groups on AM retrieval probabilities across life periods (i.e., Recent and Remote). Bivariate regression was performed to evaluate correlation between measures of AM recall and participant age, and inter-feature relationships. ANOVA was performed to evaluate changes in subject demographics across test types and AM content across participant groups and life periods. Results were corroborated by using a generalized estimating equation approach (Davis, 2002); all conclusions were equivalent with those reported using a general linear model. Where applicable, for robustness analyses (i.e., analysis across genders, native languages, and testing conditions), subject age, and/or cuing and scoring order were assigned as covariates to control for variation in the outcome variable explained by these sources. Chi-square analysis with Yates correction was run to assess group differences in the proportion of AMs assigned to multiple life span bins. Cohen’s *d* was calculated for each comparison to estimate effect size. Statistical significance was interpreted using the criterion of *p* < 0.05. False discovery rate correction was applied to multiple comparisons (Benjamini and Hochberg, 1995). Statistical analyses were performed using SPSS (IBM), Excel (Microsoft), and R (Dalgaard, 2008).

## Results

### AM Retrieval Probabilities are Modulated by Subject Age and Life Period

**FIGURE 1:**
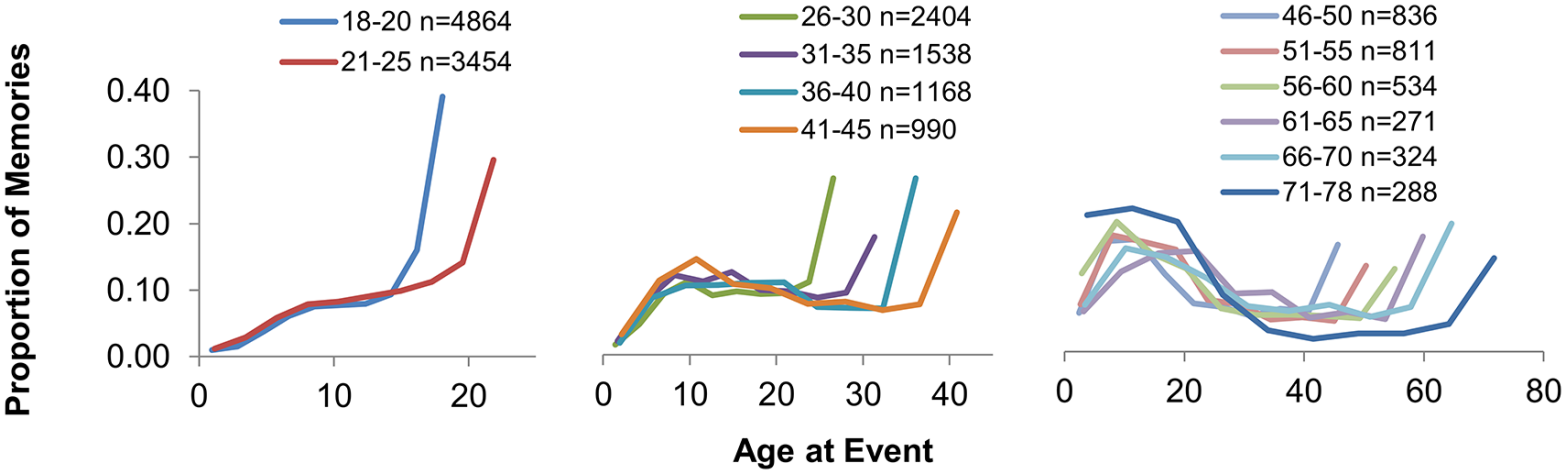
**Temporal distributions of autobiographical memories (AMs).** Participant age at the time of a recalled event was computed as the midpoint of its assigned temporal bin (see Materials and Methods); the proportions of AMs retrieved across the life span are plotted according to subject age at the time of the event (*n*: number of dated AMs). Younger subjects displayed the greatest retention of recent AMs, which gradually decreased with increasing age. The reminiscence bump (an increase in AMs recalled from adolescence to early adulthood) emerged in subjects in their mid-to-late 20s and was most prominent in subjects older than 45 years old. Childhood amnesia (a paucity of AMs recalled from the first few years of life) was clearly observed in younger subjects (18–45 years old). Its absence in older subjects (46–78 years old) is likely due to our dating procedure, as the first 10th of life in this age group is longer than the typical interval of childhood amnesia.

Autobiographical memory retrieval probabilities were analyzed against the age of the subject at the time of the recalled episode among various age groups (**Figure 1**). We observed a large proportion of AMs recalling recent events, which declined steeply with time from the present moment (retention interval). We also found a relative increase in the number of AMs dated to adolescence through young adulthood (the bump), and a relative absence of AMs from early childhood (childhood amnesia).

Moreover, these characteristics of the temporal distribution of AMs produced by CRAM changed with participant age. Retention of Recent AMs was strongest in younger subjects (18–25 years old) and decreased with increasing participant age (*p* < 0.001). For example, AMs dated to the most recent 10 years of life comprised ∼77% of all AMs cued from 18 to 25 year old subjects, ∼42% of those cued from 26 to 45 year old subjects, and ∼19% of those cued from subjects older than 45 years (see **Table 2**). Furthermore, while absent in the two youngest age groups, the reminiscence bump emerged in subjects in their mid-to-late 20s, and was most evident in subjects older than 45 years of age (**Figure 1**). The peak of the bump corresponded to the years between ages 8 and 22, depending on the age group (**Figure 1**). Age ranges presented in each panel in **Figure 1** reflect the changing clarity of the reminiscence bump quantified as the ratio of the peak retrieval probability across life span bins (excluding those associated with the retention interval) to the subsequent minimal retrieval probability. On average, this ratio is undefined in 18–25 year olds (due to the lack of a minimum, reflecting the absence of a bump), greater than one in subjects 26–45 years old, and greater than two in adults older than 45. Childhood amnesia was observed in younger subjects (18–45 years old) as demonstrated by a notable drop in AMs dated to the first 10th of the life span (less than 2%). Its apparent absence in older subjects (i.e., 46–78 years old; **Figure 1**) is likely an artifact of our methodology. Specifically, the first tenth of life of an older individual extends beyond the relatively narrow temporal period associated with childhood amnesia, and thus limits our ability to isolate and analyze memory for these very early life events.

**Table 2:** Autobiographical memory content and retrieval across life periods and age groups.

		Remote			Recent (10 years)		
		18–25 years old	26–45 years old	46–78 years old	18–25 years old	26–45 years old	46–78 years old
Total content	Mean	19.05	20.68	24.23	21.04	22.17	27.46
	SD	11.70	14.83	16.07	12.23	15.30	18.02
	CV	0.61	0.72	0.66	0.58	0.69	0.66
	Median	16	17	21	19	19	23
	IQR	13	17	18	15	16	23
AMs dated	*n* (%)	1890 (23%)	3549 (58%)	2469 (81%)	6428 (77%)	2551 (42%)	595 (19%)
AMs scored	*n*	826	1592	1016	1662	779	162

*Recent AMs are those dated to the most recent 10 years; all remaining AMs are Remote (SD, standard deviation; CV, coefficient of variation; IQR, inter-quartile range; *n*, number of AMs within each subgroup).*

### Reported AM Content is Moderately Increased in Older Adults

**FIGURE 2:**
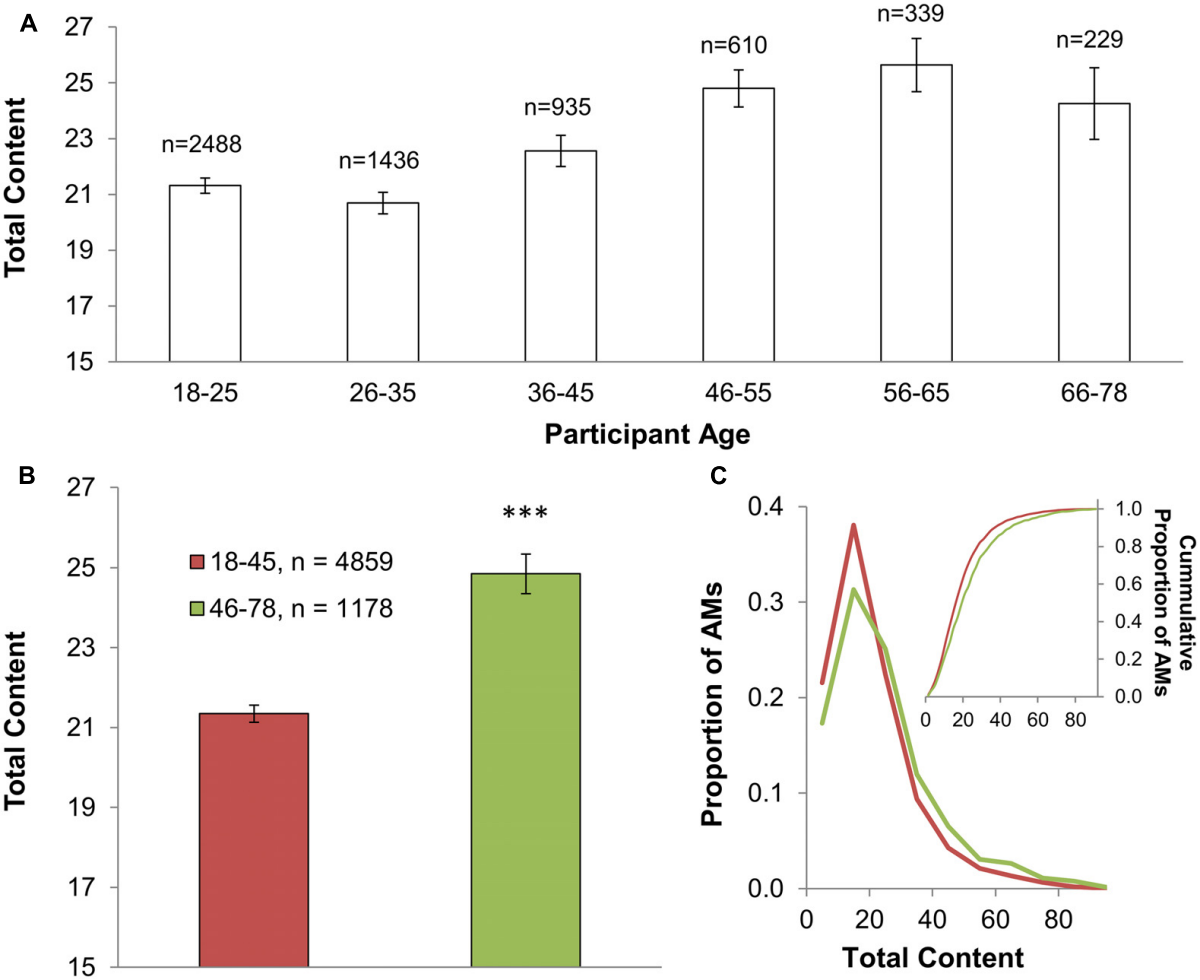
**Moderate increase of total reported content in older individuals.** The total number of unique details reported for a given AM is displayed for various age groups (*n*: number of scored AMs; error bars: SEM; ^∗∗∗^*p* < 0.001). **(A)** Total content moderately increased with the age of the participant, most noticeably in subjects older than 45 years old, an effect that persisted into old age. **(B)** The magnitude of these effects is illustrated by pooling AMs from those subjects older than 45 years old, and from those 45 years old or younger. This grouping revealed a ∼16% age-related increase (∼4 elements) in total reported content. **(C)** Distributions of these two age groups show a mild right-tail shift.

A total of 6,037 scored AMs were analyzed for content (**Table 1**; see Section “Materials and Methods” for data screening procedures). On average, subjects reported ∼22 elements (SD = 14) per AM. We found a mild yet significant positive relationship between total reported content and participant age (*r* = 0.11, *p* < 0.001). To further investigate this finding, memories were separated into six age groups (see **Figure 2A**). Upon comparison to the youngest age group (18–25 years old: Mean Total Content = 21.3, SD = 12.1), adults older than 45 years old reported a greater number of remembered details (46–55 years old: *M* = 24.8, SD = 16.3, *d* = 0.24; 56–65 years old: *M* = 25.6, SD = 16.1, *d* = 0.30; 66–78 years old: *M* = 24.3, SD = 16.8, *d* = 0.20); in contrast, reports of content from individuals younger than 46 years old (26–35 years old: *M* = 20.7, SD = 14.0; 36–45 years old: *M* = 22.6, SD = 16.4) were found to be comparable to those collected from the youngest group (*d* = 0.05, and *d* = 0.09, respectively). Collectively, these findings suggest a non-linear effect of age on total reported content; the age-related increase in the amount of detail reported in typical autobiographical recollection emerges most noticeably in subjects in their mid-to-late 40s and persists into old age (i.e., late 70s; **Figure 2A**). Given this pattern, to simply describe the magnitude of these effects, AMs were divided into two groups: those collected from subjects 45 years old or younger (Mean Age = 27, SD = 8, range: 18–45 years old; 72% female, 80% native English speakers) and those from subjects 46 years old or older (Mean Age = 57, SD = 8, range: 46–78 years old; 65% female, 89% native English speakers; **Figure 2B**). This grouping revealed a ∼16% increase in the number of reported details for a given AM in older compared with younger subjects (∼25 vs. ∼21; *p* < 0.001, *d* = 0.23; **Figure 2B**; **Table 2**). While reported content was quite variable from memory to memory, the coefficient of variation was similar across all ages (∼0.6–0.7; **Table 2**). The distributions of total content underscore the moderate effect of age on reported details (**Figure 2C**). Half of all scored memories in younger subjects were comprised of ∼16 or fewer elements; half of all scored AMs in older subjects contained more than ∼20 elements.

### Total Content Decays with the Age of the Memory Across Younger and Older Subjects Alike

**FIGURE 3:**
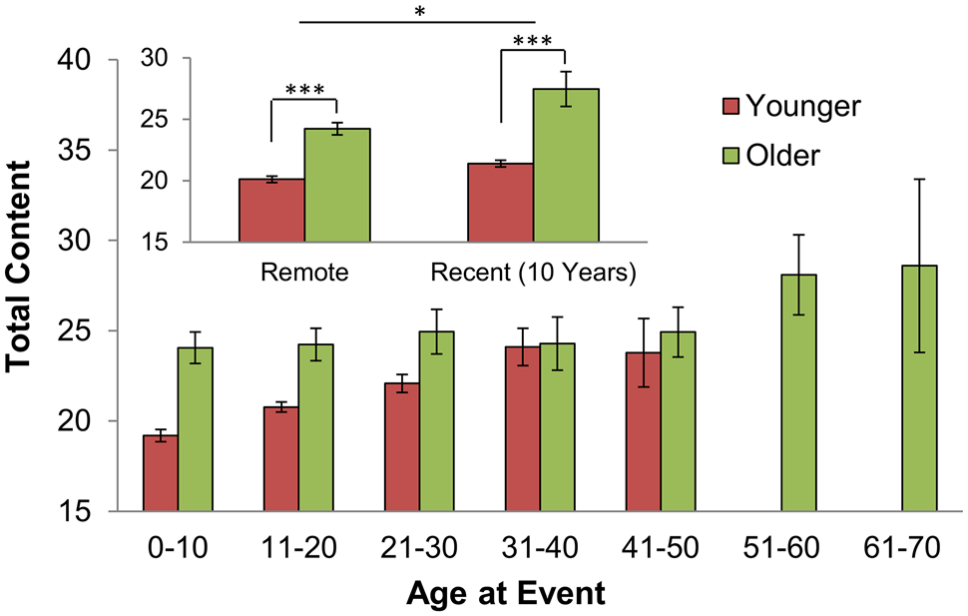
**Reported details from Remote and Recent AMs across age groups.** Older individuals reported a greater number of total details from each decade of life (Younger subjects: 18–45 years old; older subjects: 46–78 years old; error bars: SEM). Inset: Recent AMs are those dated to within 10 years of the present; all remaining AMs are Remote (^∗^*p* < 0.05; ^∗∗∗^*p* < 0.001; see **Table 2** for sample sizes within Remote and Recent intervals). Content declined with the age of the episode among all subjects.

Older subjects reported significantly more content than younger subjects for memories of all life periods, both when comparing equivalent decades of life (**Figure 3**), and when comparing relative life periods (e.g., Recent: the most recent 10 years, and Remote: > 10 years from the present; see Materials and Methods; **Figure 3** Inset; **Table 2**). The numerical results of content analysis of Recent and Remote AMs only marginally fluctuated depending on how these temporal intervals were defined (e.g., restricting Recent AMs to the most recent 5 years; restricting Remote AMs to the first decade of life). In addition, all reported conclusions remained unchanged and did not depend on the particular grouping applied (data not shown).

Older subjects reported ∼24 elements from AMs dated to the first two decades of life compared with ∼20 elements in younger subjects (*p* < 0.001; *d* = 0.27). Likewise, Recent AMs from older subjects were comprised of ∼27 elements compared with ∼21 elements in those from younger individuals (**Figure 3** Inset; *p* < 0.001; *d* = 0.38). Total content declined with the age of the episode among all age groups. For example, Remote AMs were comprised of significantly fewer details compared with Recent AMs (*p* < 0.01; **Table 2**; **Figure 3**). These results confirm those previously reported in college-aged subjects (Gardner et al., 2012) and extend these findings to older adults.

### Features Selectively Contribute to the Age-Related Increase in Total Content

**FIGURE 4:**
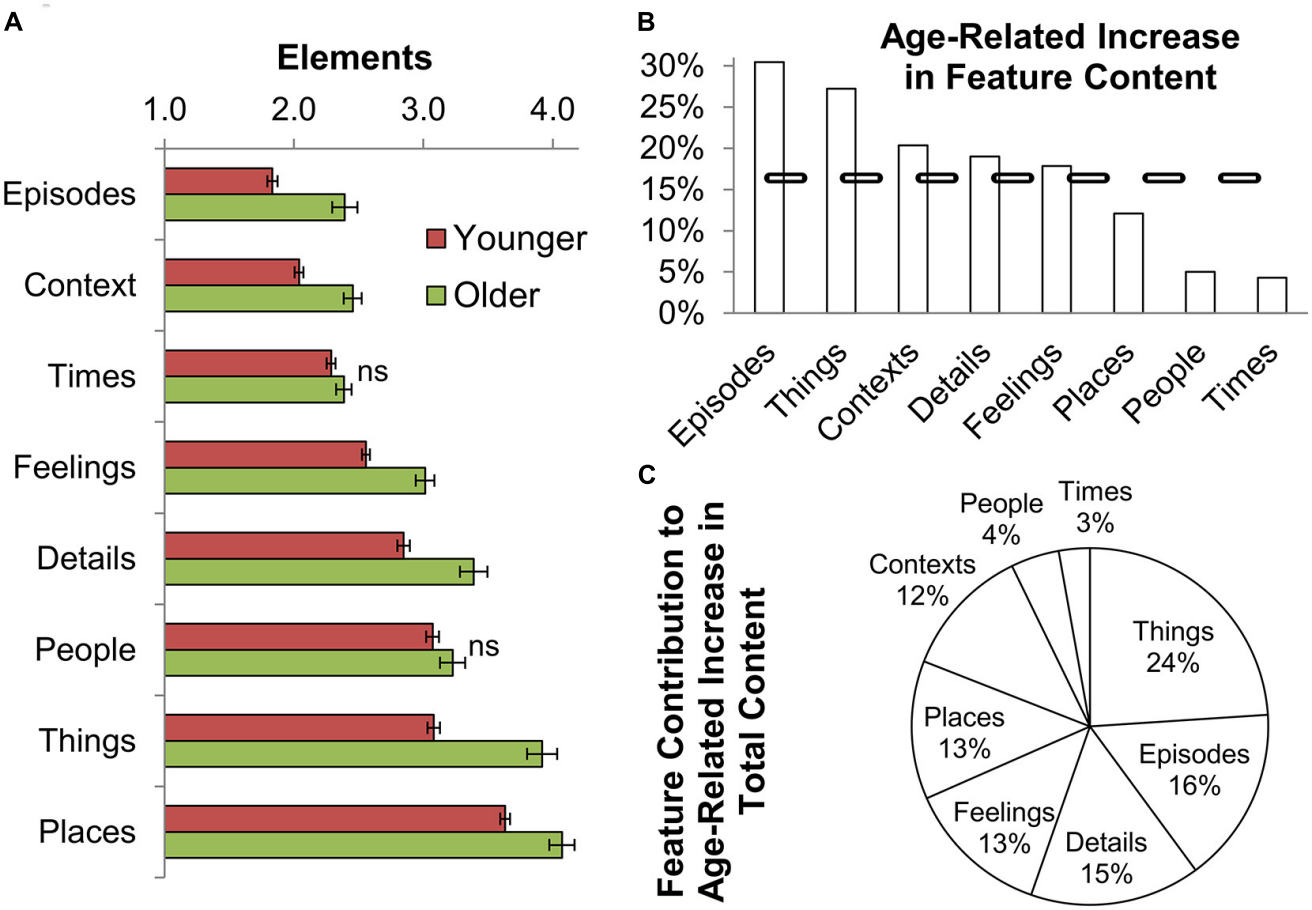
**Selective feature contribution to the age-related increase in total content. (A)** Subjects of all ages reported more AM elements related to *Places*, *Things*, and *People*, and fewer related to *Episodes, Contexts*, and *Times* (Younger subjects: 18–45 years old; older subjects: 46–78 years old). Older adults reported more elements within all features (the age-related increase is statistically significant unless marked as *ns*; error bars: SEM). **(B)** *Episodes* and *Things* showed the most prominent age-related increase, as *People* and *Times* stayed close to that observed in younger adults. The dashed line indicates the overall age-related increase in total content (∼16%). **(C)** The observed increase in total reported content from younger to older subjects (∼4 elements; see **Figure 2**) was largely composed of *Things, Episodes*, and *Details*.

A relatively high proportion of AM content (∼46%; ∼10.1 elements) was associated with *Places, Things*, and *People*. In contrast, *Times*, *Contexts*, and *Episodes* were less represented, together comprising just ∼29% (∼6.4 elements). *Details* and *Feelings* were close to the average at ∼13% and ∼12%, respectively (**Figure 4A**). Largely in line with these distributions, the proportion of AMs containing at least one element of a particular feature was highest for *Places* (98%) followed by *People* and *Feelings* (92% each), *Things* (89%), *Details* (84%), *Times* (83%), *Contexts* (78%), *and Episodes* (63%). Feature variation was higher than that observed for total content, but equivalent across age groups (*Mean Feature CV* = 1.0). All these findings uphold those previously reported in younger subjects (Gardner et al., 2012).

Adding to this research, we found that older adults reported a greater number of elements among all features. *Episodes* and *Things* showed the most prominent age-related content increase (∼30% and 27%, respectively; *p* < 0.001), while content associated with *People* and *Times* remained close to that observed in young subjects (*p* > 0.10; **Figures 4A,B**). These data collectively indicate that the age-related increase in total content is not uniformly distributed across features (in terms of either absolute or relative value). In particular, *Things* (24%), *Episodes* (16%), and *Details* (15%) contributed more substantially to the age-related increase observed in total content (**Figure 4C**), whereas contributions from *People* (4%) and *Times* (3%) were definitively smaller. The proportion of AMs containing at least one element from a given feature was significantly higher in older compared with younger subjects for *Feelings, Things, Details, Contexts*, and *Episodes* (∼5% higher on average; *p* < 0.001) but not for *People, Places*, and *Times* (*p* > 0.10). These feature distributions were consistent between Remote and Recent AMs in both younger and older subjects (Supplementary Figure S1); notably, the feature *Times* appeared to be least resilient to temporal decay of content among all subjects.

### People is a Relatively Independent Feature of Recall Among All Ages

Content correlation analysis showed positive relationships among all features (*mean Pearson r* = 0.37). Moreover, these relationships were similar across age groups (younger: *r* = 0.36; older: *r*= 0.40) as well as between Remote and Recent intervals (Remote: *r* = 0.39; Recent: *r*= 0.37). To further evaluate feature dependence, the correlation between each feature and all other content was computed. The average of these values across all features was equivalent between age groups (younger: *r* = 0.53; older: *r* = 0.57; Supplementary Figure S2A) and across life periods (not shown). However, the feature *People* exhibited a comparatively mild relationship (*r* = 0.37; 32% less than the average: Supplementary Figure S2B) across all conditions, suggesting that it is a relatively independent component of recall. The inter-feature relationships found here confirm those previously reported among younger subjects (Gardner et al., 2012) and extend these findings to individuals distributed across the life span.

### Estimates of Retrieved Content across Age Groups and Life Periods

**FIGURE 5:**
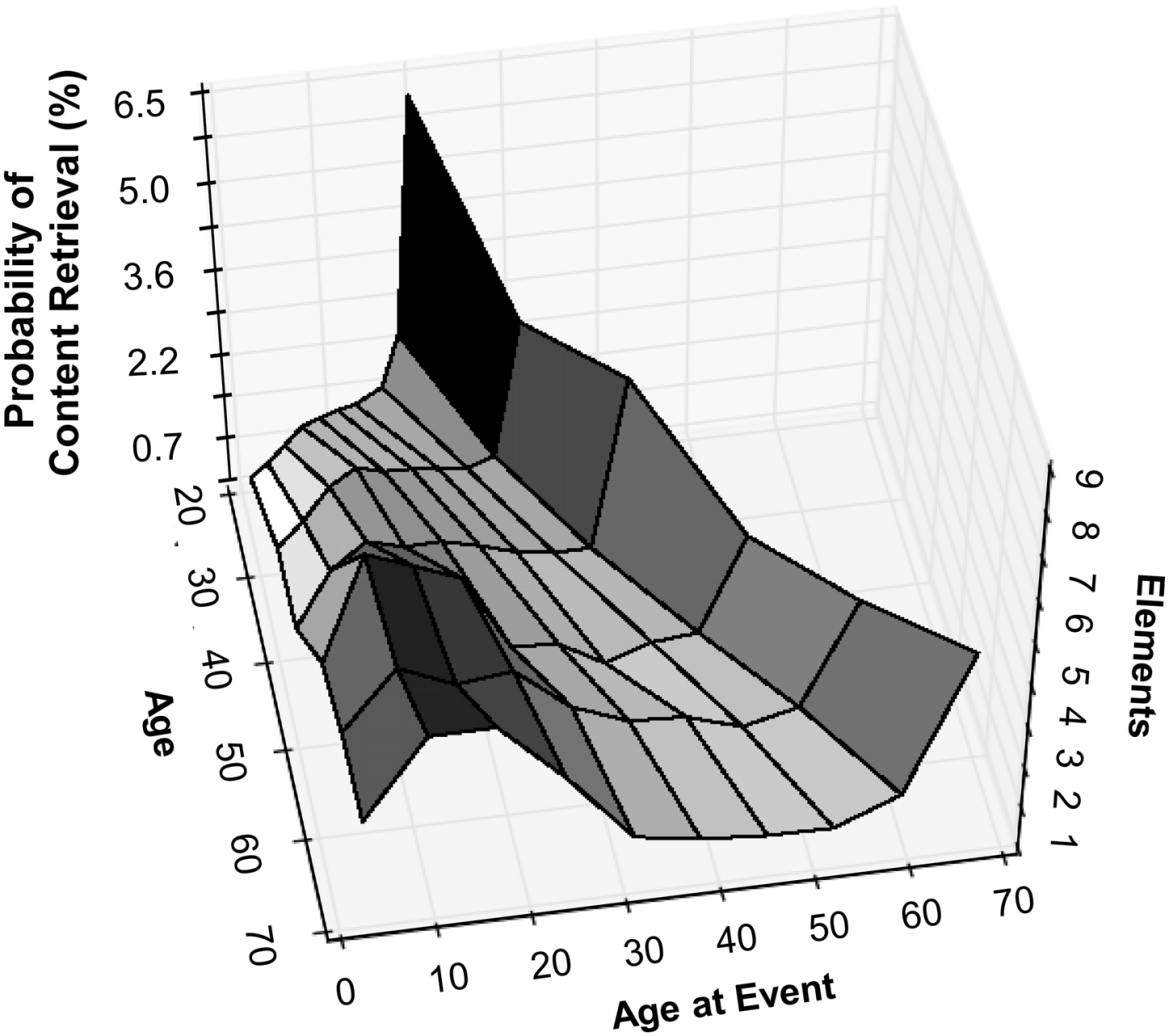
**Estimated distribution of retrieved content across age groups and life periods.** Content measures within each temporal bin for each age group were normalized to their applicable retrieval probability. In particular, the number of elements per life period was computed for each of the six age ranges outlined in **Figure 2A** (and plotted here by mean age within each group). These same computed values (normalized to all data points) provide estimates of the likelihood that any given amount of retrieved content is associated with a particular age and life period (shown as content retrieval probabilities).

This work provides numerical description of AM retrieval probabilities and reported content associated with distinct life periods. Combining these two measurements, we can estimate the relative distribution of retrieved content across temporal intervals and age groups (**Figure 5**). Specifically, given the number of elements typically reported for a single retrieved AM, for each of the various age groups (as outlined in **Figure 2A** and plotted in **Figure 5** by mean age), we computed a probabilistic content distribution among life periods. For example, when experiencing AM, a typical 70 year old, on average, reports a similar content amount from his or her middle teenage years compared with that from the last few years of his or her life. In contrast, content from events dated to the most recent 2 years of the life of a 21 year old is ∼5 times more represented in memory than that associated with events from his or her middle teenage years. Assuming that the frequency of AM recollection is stable across age groups (e.g., see Gardner and Ascoli, 2015), we can further estimate the relative probability that a particular recalled element is associated with a certain age group and life period. For example, the likelihood for a 30 year old to retrieve content from his or her early 20s (∼1.3%) is equivalent to that for a 60 year old to retrieve content from his or her early thirties.

### The Bump is Unrelated to Changes in Total Content and Feature Content

Autobiographical memories found within the reminiscence bump may have distinct recall characteristics. For example, AMs that compose the bump may be comparatively rich with recalled detail. Such a finding would explain, at least in part, why this life period plays a particularly prominent role in subjective experience. We evaluated reported content from AMs within and beyond life periods associated with the bump in older subjects (46–78 years old) for whom this phenomenon was most pronounced. In this age range, we observed a relatively high AM retrieval probability (excluding temporal bins associated with the retention interval) from ages 11–20 and a relatively low probability from ages 31–40 (see **Figure 1**). However, measures of total content from these life periods did not significantly differ (*M* = 24.90 and *M* = 25.28, respectively; *p* > 0.10, *d* = 0.02; **Figure 3**). The composition of memories dated to these life periods was also quite stable (*p* > 0.10), with any given feature showing a deviation of ∼2% or less (*Mean deviation*: ∼1%).

All the main findings of this work are robust to gender, native language, and changes in experimental procedures (see Supplementary Tables S1 and S2). For example, total reported content moderately increased with age both for females and males, as well as for native and non-native English speakers (see Supplementary Material). However, several minor quantitative distinctions in AM recall were observed between males and females, between native and non-native English speakers, and among testing conditions. A full review of these results is included as Supplementary Material.

### Age Effects are Corroborated Using Subject As the Level of Analysis

As prior reports suggest that different individuals have distinct recollection experiences (Rubin et al., 2004), the main conclusions of this work were evaluated using a subject-level analysis. Given the relatively large amount of variability in content scores from memory to memory (see **Table 2**), the analysis was restricted to those subjects who scored five or more memories (18–45 years old: *n* = 496; 46–78 years old: *n* = 118). This restriction permitted measures that more closely represent typical recollection from a given individual. In addition, for inclusion in comparisons across Recent and Remote intervals, subjects were required to have an AM scored in each temporal period (18–45 years old: *n* = 420; 46–78 years old: *n* = 69). Content measures reported independent of temporal periods were weighted according to the temporal distribution of AM retrieval computed separately for each subject on the basis of his or her dated AMs.

Age effects on the temporal distribution of AMs and AM content are confirmed by subject-level analysis. In particular, the proportion of AMs from Recent life intervals was relatively high in young subjects (18–25 years old: ∼77%) and decreased with increasing age (26–45 years old: ∼40%; 46–78 years old: ∼17%). Moreover, older adults (46–78 years old) reported ∼15% more elements than younger (18–45 years old) subjects (Mean ± SD: 25 ± 13 compared with 21 ± 10 details; *p* < 0.01), replicating age differences found using memory as the observational unit. Remote memories contained fewer elements (18 ± 11) than Recent memories (23 ± 13; *p* < 0.001) across all subjects, and older compared with younger subjects reported more elements from Recent (Older subjects: 29 ± 18; Younger subjects: 22 ± 11; *p* < 0.001) and Remote events (Older subjects: 23 ± 13; Younger subjects: 18 ± 10; *p* < 0.001). In addition, an age-related increase in total reported content was most drastic for Episodes (∼31%) and Things (∼30%), whereas considerably less dramatic for Times ( < 1%) and People (∼4%), closely resembling findings using memory as the unit of analysis. Likewise, total content scores from older subjects did not discriminate between bump and non-bump memories (e.g., comparing content from AMs dated to when subjects were 11–20 years old with those dated to when subjects were 31–40 years old; *p* > 0.10; data not shown).

## Discussion

This work provides quantitative measures of reported AM content from individuals that represent a substantial segment of the adult population (18–78 years old). This was accomplished using CRAM, an instrument designed to collect counts of details that fall within specified features from naturalistically elicited AMs dated to particular life periods. Relying on participant counts of memory content (rather than experimenter-scored participant narratives) facilitates data collection and thus enables fine-scale analysis of AMs (e.g., as shown in **Figure 5**).

We previously demonstrated that CRAM replicates several noted observations of AM recall (e.g., on the retention of recent AMs and their associated content; Rubin and Schulkind, 1997; Levine et al., 2002; Piolino et al., 2002; Janssen et al., 2011). The present study upholds our previous findings from college-aged subjects. In particular, the current estimate of AM total content (∼21 elements) among younger subjects is almost identical to that reported (∼20 elements) by Gardner et al. (2012). In addition, the current work supports previous findings on the temporal decay of content (Remote AMs contain fewer elements), AM content variability (from memory to memory, total content remains relatively stable compared to feature content), AM composition (*Places, Things, and People* are prominent features of recall), and inter-feature correlations (*People* is a relatively independent recall characteristic).

Extending CRAM to older adults, we replicated prior reports of age-modulation of the temporal distribution of AMs. Specifically, Recent AMs were considerably less likely to be recalled in older subjects (Rubin and Schulkind, 1997; Janssen et al., 2011), and the reminiscence bump emerged in subjects in their mid-to-late 20s. While some studies have found that the bump is not apparent until ∼40 years of age, using relatively small temporal bins, Janssen et al. (2011) reported a similar onset to that found here. In addition, the temporal interval associated with the bump using CRAM (i.e., 8–20 years old) is consistent with these studies.

Total content moderately but significantly increased with subject age. Older adults reported ∼25 details for a given AM, ∼4 elements more than the number reported from younger subjects. Moreover, this age-related increase in content was observed for Recent and Remote memories, and was most drastic for the features *Episodes* (sequences of event) and *Things* (objects) while negligible for *People* (unique individuals) and *Times* (temporal detail). Altogether, these data quantitatively describe an age-associated shift in the reported details of subjectively remembered events.

As older adults reported more content than younger adults from memories that originated in the same decade of life (i.e., those AMs that have similar ages of encoding), these findings appear to highlight the re-constructive nature of AM (Bartlett, 1932; Conway and Pleydell-Pearce, 2000; Hupbach et al., 2007). This interpretation assumes that the initial number of encoded event details and encoding depth are similar between age groups. However, as with all cross-sectional aging research, any inter-group differences may reflect generational differences rather than (or in addition to) changes that occur among individuals across their life span.

It also remains possible that older and younger adults used divergent strategies to establish feature counts. However, the finding that the age effects on content were feature-specific (see **Figure 4**) argues against a general change in interpretation of CRAM’s instruction, and/or adjustment in content evaluation (e.g., a pervasive tendency for older individuals to report higher scores). Similarly, older subjects may be more motivated to recall and report event details. Nevertheless, the selection of online test types (which differed in their time commitment) was equivalent across age groups, suggesting that motivation may not underlie our findings. A similar conclusion can be drawn from the finding that content data collected in-person were equivalent to those collected online (see Supplementary Material), assuming that, on average, testing conditions correlate with task motivation. Further work, including the use of longitudinal designs, is required to clarify the mechanisms underlying the current findings.

Older individuals are proposed to have altered narrative attention and to tell more interesting life stories (James et al., 1998). Although more detail is not always better, inclusion of information about sequences of happenings (the feature that was most strongly augmented from younger to older subjects) within a life narrative enables placement of an episodic snapshot into a broader context of surrounding events (and may enhance storytelling). It would be interesting to establish how our findings on feature-specific age-related modulation of reported AM content compare with the types and amount of detail shared through social communication of event memories and during the narration of life stories.

Despite the moderate change in total reported content, several properties of recollection were stable across age groups. In particular, AMs from younger and older subjects, and those from Remote and Recent life periods, showed similar feature distributions. In addition, among all ages, fewer details were reported from Remote than from Recent AMs. Thus, these data are indicative of two independent age effects on reported AM content: a positive correlation with the age of the individual and a negative one with the age of the event. These findings confirm and extend prior studies (Janssen et al., 2011; Gardner et al., 2012).

Additionally, independent of age, almost all AMs were reported to have at least one detail related to location, and nine out of 10 memories were reported to include some information about people, objects, and feelings, suggesting that these features are quite remarkable and/or at the core of subjective recall. In contrast, less than two-thirds of memories were reported to include sequential events. The feature *People* was also relatively independent, further demonstrating its unique role in memory; among the proposed functions of AM, remembrance of the individuals associated with specific life experiences is essential to form and maintain social relationships (see Pillemer, 1992; Bluck et al., 2005; Bluck and Alea, 2011; Waters, 2014).

Combining the observed temporal distributions of AM retrieval with measure of reported AM content permits computation of detailed probability estimates of reporting an element from a given life period at a particular age (**Figure 5**). For instance, this approach quantifies how likely it is for a detail retrieved by a 60 year old to be associated with an episode from his or her 20s (∼1.7%). Moreover, we can address questions on how these probability distributions change with subject age. For instance, how does the previously computed probability compare with the likelihood that the same amount of content retrieved by a 20 year old stems from a relatively recent event?

Several factors are proposed to account for the high accessibility of memories that compose the reminiscence bump, e.g., neurocognitive development, cultural influence, and life span changes in encoding efficiency (Rubin et al., 1998; Schrauf and Rubin, 2000; Berntsen and Rubin, 2004; Rathbone et al., 2008; Bohn and Berntsen, 2010; Janssen et al., 2015). We add to an understanding of the bump by showing that neither reported counts of detail across all features nor counts within individual features explain or are explained by the relatively high probability of recollection associated with this life period. These data are in line with previous studies that have demonstrated that AMs within the bump do not have higher ratings of certain characteristics of recollection (e.g., vividness, rehearsal, reliving, novelty, emotionality; Rubin and Schulkind, 1997; Janssen et al., 2011).

Past approaches reporting counts of AM content have predominately focused on the distinction between episodic and semantic retrieval (Levine et al., 2002; Piolino et al., 2002; Addis et al., 2008, 2010; also see Tulving, 1972, 1985). Episodic memory recounts a unique personally experienced event, with some form of contextual information (e.g., spatiotemporal detail). Semantic memory recalls abstracted knowledge of the world or of oneself (generally acquired from repeated experiences) that does not describe or call to mind a unique episode. This distinction is highlighted by case reports of neurocognitive deficits following targeted brain damage (Rosenbaum et al., 2005) and is present in numerous theories of AM (e.g., Conway and Pleydell-Pearce, 2000). Using a narrative scoring technique of event-cued AMs specified to life periods, Levine et al. (2002) found that, compared to younger subjects, older adults report fewer episodic but a similar or greater number of semantic details from typical memories. This age effect on episodic detail appeared to be feature-dependent as it was absent (but never reversed) for some features (e.g., *Times*). Contrasting accounts, however, have been reported. Hashtroudi et al. (1990) found that older adults reported more content for feelings and thoughts (albeit these same individuals showed a reduction in sensory-perceptual recollection). In addition, Janssen et al. (2011) found that ratings of AM vividness and re-living, proposed indices of episodic remembering were higher in older subjects (also see Rubin and Schulkind, 1997; Rubin and Berntsen, 2009). Direct comparison of memory content scores obtained using a variety of quantitative and qualitative approaches will be a useful endeavor to reconcile the apparently discrepant findings in AM recollection across age groups.

We emphasize that CRAM was broadly designed to measure the details that a participant considers part of an AM, i.e., the subjective content associated with a remembered life event. As such, CRAM does not classify reported elements as episodic or semantic. However, each feature definition was worded to collect the details that compose a memory for a temporally specific event; likewise, the general guidance provided to subjects emphasized reporting of detail unique to the specified episode (see Materials and Methods; Gardner et al., 2012). As CRAM collects reports of retrieved subjective detail, this approach also contrasts with those that aim to collect “true” or verifiable detail or those which collect all potentially retrievable details associated with an event (e.g., Mello and Fisher, 1996; Levine et al., 2002). We further stress that as counts of memory details are provided by the subject, although the results are in line with several prior findings, content measures as reported here may systematically differ from the actual numbers of details that are successfully retrieved from a given event memory. As CRAM’s instruction was identical between all subjects, however, relative measures between and within age groups should reflect genuine changes in subjective memory.

Altogether, the data presented here provide previously inaccessible fine-scale quantitative characterizations of the reported subjective content of AMs as a function of the age of an individual and the age of a memory. These characterizations point to a moderate but significant age-associated feature-specific shift in how one’s life story is perceived and recounted.

## Conflict of Interest Statement

The authors declare that the research was conducted in the absence of any commercial or financial relationships that could be construed as a potential conflict of interest.

## Footnotes

1 http://cramtest.info

2 http://cramtest.info

3 http://www.natcorp.ox.ac.uk

4 http://cramtest.info

5 http://cramtest.info
