# Supplementary material for: Older adults report moderately more detailed autobiographical memories
Source: Front Psychol. 2015 May 19;6:631. doi: 10.3389/fpsyg.2015.00631 (PMC4436582; doi:10.3389/fpsyg.2015.00631)

Supplemental Information

AM retrieval and reported content are largely robust to participant subgroups and testing conditions.

All the main findings of this work are robust to gender, native language, and testing condition. Similar to the overall findings, each participant group (i.e., males, females, native English speakers, and non-native English speakers) showed age-related modulation of life span retrieval probabilities and age-related augmentation of total reported content (Tables S1-S2). The distribution of features that composed a typical AM was stable across all of these groupings (plus or minus less than 2% for any given feature), as were feature correlations (data not shown). In addition, Internet-collected AMs from younger and older groups were equally distributed among test types (see Methods; Table 1), and showed similar measures of recall to those collected in-person.

Nonetheless, several minor differences in AM recall were observed across participant groups and testing conditions. By and large AM retrieval and reported content were equivalent across genders and native languages (Tables S1-S2). However, among subjects aged 26-45 yo, we observed an increase in the number of AMs dated to Remote life periods from native English speakers (61%; compared to non-native English speakers: 47%; $p < 0.001$). In addition, the number of reported elements was increased among females (~22.8; compared to males: ~18.3; $p < 0.001$, $d = 0.31$) and native English speakers (~22.2; compared to non-native English speakers: ~18.3; $p < 0.001$, $d = 0.28$) in this same age range. The stability of feature distributions across all comparative conditions suggests that these effects are not a result of differential reporting of particular features. Nevertheless, these findings are restricted to a narrow age range (Tables S1-S2) with correspondingly small samples of scored AMs (males: $n = 738$; non-native English speakers: $n = 460$). Thus, proper interpretation of these differences will require further study of the influence of native language and gender on measures of AM retrieval and content.

All test variations produced AM temporal distributions with similar shapes and expected age-dependent modulation. Testing completed in-person, however, typically cued a greater number of AMs from Recent life periods compared with those cued over the Internet ($p < 0.001$); comparison between these conditions was restricted to young subjects (18-36 yo) as they accounted for more than 95% of the AMs collected in-person. In addition, AM cue order was inversely associated with the age of the episode (i.e., years from the present; $r = -0.22$, $p < 0.001$). Thus, tests that collected more AMs (e.g., Full compared to Mini; Mini compared to Atomic; see Methods) typically elicited a higher proportion of Recent AMs. After controlling for cuing order, however, retrieval was equivalent across all test types ($p > 0.10$).

Total content was equivalent between AMs scored in-person and online (~21.1 and ~21.5, respectively; $p > 0.10$). In addition, feature contributions to total content and inter-feature correlations were generally robust to test location, although there was an increase in reporting of *People* during in-person testing (~13% of content was comprised of *People* in testing done online compared to ~17% when completed in-person). Among Internet-scored AMs, the Full test was associated with content scores lower than those produced by all other test types (Full: $M = 20.1$, $SD = 15.4$; All other Tests: $M = 24.6$, $SD = 14.4$, $d = 0.30$), an effect that appears to be

driven by younger subjects. Fatigue potentially associated with longer tests did not underlie this finding, as scoring order did not affect reported content ($p > 0.10$). Since the test options were self-selected, it is plausible that those individuals who chose to complete the Full test vary systematically in the number of elements they typically retrieve, and/or how they interpreted CRAM's instruction. Alternatively, subjects who are more likely to randomly score AM content may be more likely to select specific test types. The finding that AM feature composition and inter-feature correlations were equivalent between all test types ($p > 0.10$), however, suggests otherwise. Altogether, variations in retrieval probabilities and AM content reported here add to the AM research literature showing that mild alterations in testing conditions can have statistically significant effects on outcome measures (e.g., as shown by demand characteristics and their effect on the temporal distribution of AM; Rubin and Schulkind, 1997).

Table S1. AM retrieval probabilities and content across genders. Measures of AM content (mean; standard deviation: SD; coefficient of variation: CV; median; inter-quartile range: IQR) showed an age-related increase among females and males alike. Both genders similarly showed an age-dependent decrease in the proportion of AMs dated to a Recent temporal interval (within ten years from the present). In subjects 26-45 yo, however, AMs from females (compared to males) were associated with a greater number of reported details. The number of AMs (*n*) within each subgroup is presented.

		Female			Male		
		18-25 yo	26-45 yo	46-78 yo	18-25 yo	26-45 yo	46-78 yo
Total Content	Mean	21.48	22.82	24.43	20.78	18.34	25.60
	SD	12.01	15.22	16.37	12.36	14.03	16.36
	CV	0.56	0.67	0.67	0.59	0.76	0.64
	Median	18	19	21	17	14	22
	IQR	14	17	17	15	15	21
AMs Dated	*n*	6174	3929	2036	2144	2171	1028
	(% Recent)	(77%)	(40%)	(20%)	(77%)	(46%)	(18%)
AMs Scored	*n*	1895	1633	786	593	738	392

Table S2. AM retrieval probabilities and content across native and non-native English speakers. Measures of AM content (mean; standard deviation: SD; coefficient of variation: CV; median; inter-quartile range: IQR) showed an age-related increase among native and non-native English speakers alike. Both groups similarly showed an age-dependent decrease in the proportion of AMs dated to a Recent temporal interval (within ten years from the present). In subjects 26-45 yo, however, AMs from native English speakers (compared to non-native English speakers) were associated with a greater number of reported details and were more likely dated to Remote life periods. The number of AMs (*n*) within each subgroup is presented.

		English			Non-English		
		18-25 yo	26-45 yo	46-78 yo	18-25 yo	26-45 yo	46-78 yo
Total Content	Mean	21.07	22.19	24.98	22.11	18.27	23.83
	SD	11.83	15.60	16.58	12.98	11.67	14.62
	CV	0.56	0.70	0.66	0.59	0.64	0.61
	Median	17	18	21	19	15	21
	IQR	13	17	19	15	13	18
AMs Dated	*n*	6439	4683	2738	1879	1417	326
	(% Recent)	(77%)	(39%)	(19%)	(78%)	(53%)	(21%)
AMs Scored	*n*	1959	1911	1044	529	460	134

Figure S1. AM composition is equivalent across Remote and Recent periods. Remote and Recent AMs were similarly composed of individual features among **(A)** younger (18-45 yo) and **(B)** older (46-78 yo) subjects. In addition, feature distributions of AM content among Remote and Recent intervals were equivalent to those observed overall (compare to Fig. 4 in main text).

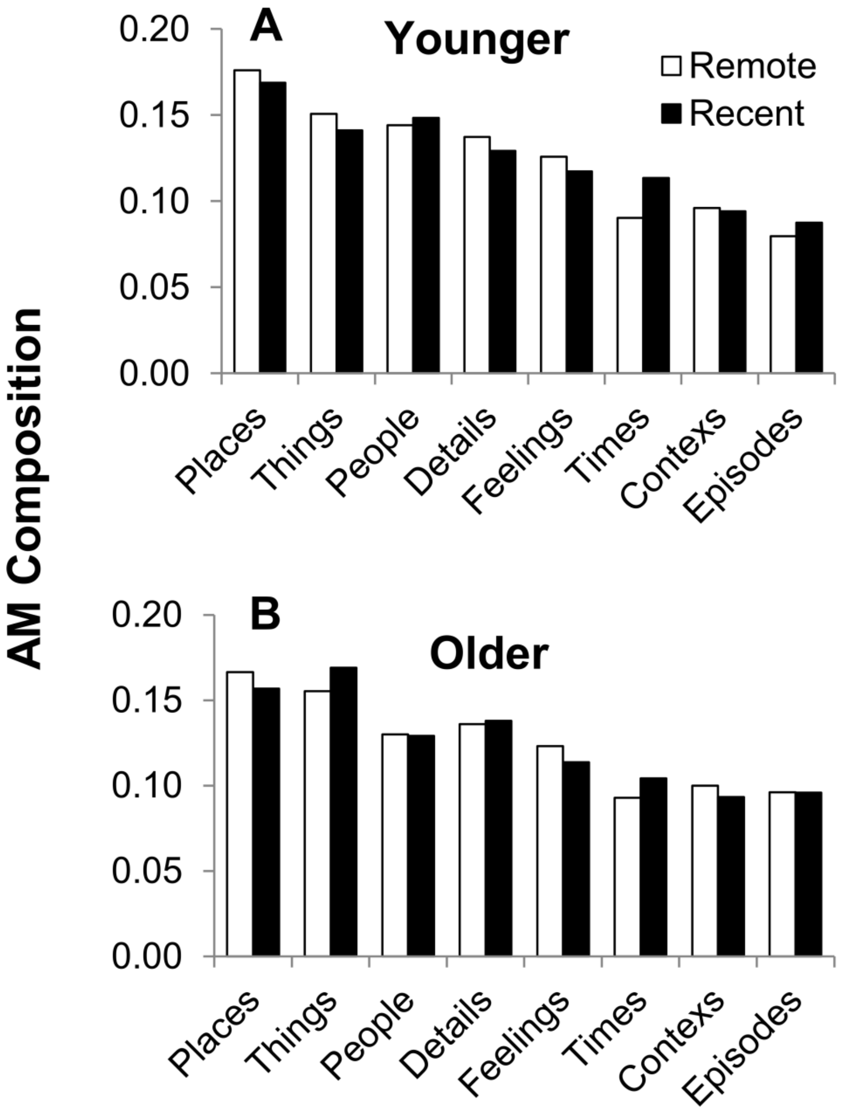

Figure S2. People is a relatively independent feature of recollection. To assess feature independence, correlation between content from a given feature to content from all remaining features was computed. **(A)** The mean of these values across all features was equivalent among younger ($r = 0.53$) and older ($r = 0.57$) subjects. **(B)** Moreover, among all ages, People appeared to be a relatively independent feature of recall (32% less than the mean); values shown for a given feature were normalized to the mean (dashed line) across features for the applicable age group. Values represent Pearson correlation coefficients. All correlations are statistically significant ($p < 0.001$).

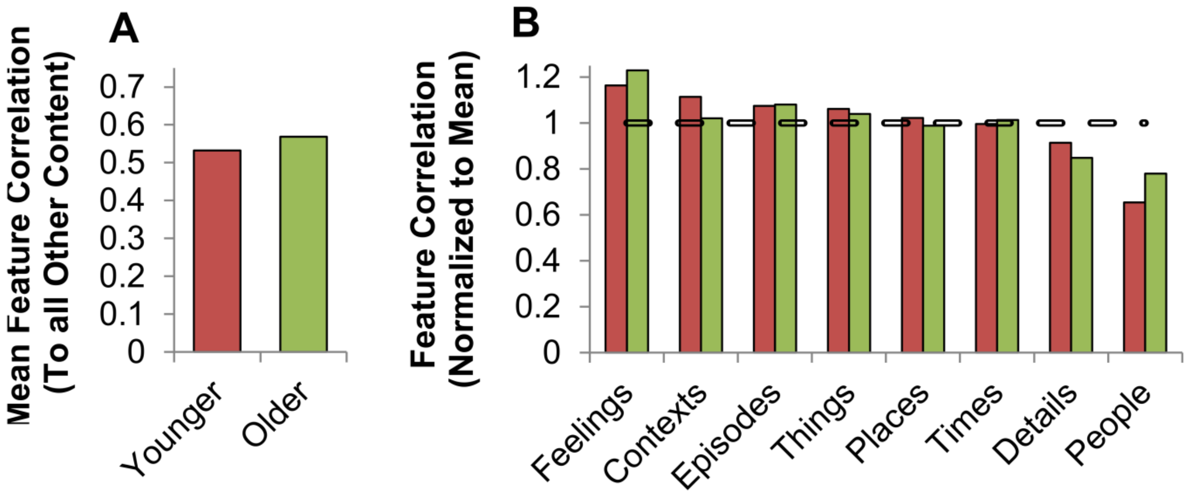

Supplement: Supplementary file 1 [file Data_Sheet_1.PDF]
